# Self-Healing Performance of Asphalt Concrete with Ca-Alginate Capsules under Low Service Temperature Conditions

**DOI:** 10.3390/polym15010199

**Published:** 2022-12-30

**Authors:** Huoming Wang, Quantao Liu, Jie Wu, Pei Wan, Feiyang Zhao

**Affiliations:** 1 National Engineering and Research Center for Mountainous Highways, China Merchants Chongqing Communications Technology Research and Design Institute Co., Ltd., Chongqing 400067, China; 2 State Key Laboratory of Silicate Materials for Architectures, Wuhan University of Technology, Wuhan 430070, China

**Keywords:** calcium alginate capsules, asphalt concrete, selfhealing level, low service temperature

## Abstract

Calcium alginate capsules containing rejuvenators represent a promising method for asphalt concrete premaintenance, but their healing capacities under lower temperature conditions are still unknown. This paper investigated the healing performance of asphalt concrete containing calcium alginate capsules at low service temperatures. The Ca-alginate capsules were synthesized, and their morphology, compressive strength, thermal resistance, and relative oil content were evaluated. Besides, evaluations for the healing of asphalt concrete and the rejuvenator-release ratio of the capsules were determined via fracture-healing-refracture testing and Fourier-transform infrared spectrum experiments. Meanwhile, the glass transition temperature and rheological property of asphalt binder after compressive loading under different temperatures were explored via a differential scanning calorimeter and dynamic shear rheometer. The results showed that the capsules had good thermal resistance and mechanical strength. The capsules released less oil under −15, −10, and −5 °C than at 20 °C, and the healing ratios of the asphalt concrete with the capsules at −15, −10, and −5 °C were obviously lower than that at 20 °C. The released rejuvenator from the capsules could decrease the complex modulus and glass transition temperature of the asphalt binder. When compared with low service temperatures, the asphalt binder containing the capsules and serving at a high temperature has a better softening effect and low-temperature performance due to more oil being released.

## 1. Introduction

Asphalt mixtures represent the leading surfacing material for roads and bridges due to excellent pavement performance. Different asphalt modification technologies were developed to enhance the road performance and service life of asphalt pavement [1]. Nonetheless, under the combined actions of cold temperature [2], ultraviolet radiation exposure [3], water erosion [4], and repetitive vehicle loading [5], microcracks will inevitably occur on asphalt concrete during its service life. Without effective treatment for the pavement, the microcrack will fatally evolve into macrocracking, which will lead to the failure of the concrete structure and, thus, shorten the lifetime of the asphalt pavement.

In order to keep asphalt pavement in a good serviceable condition, road agencies usually take maintenance measures regarding pavement. The current crack repair treatments can extend the service life of asphalt pavements to some extent, but these are conducted on pavements passively after the occurrence of structural damage, which requires considerable raw materials and imposes an additional burden on the environment [6,7,8]. Therefore, there is an urgent need for sustainable treatments in pavement maintenance in a carbon-neutral context.

Asphalt, as a natural healing material, can recover its original properties spontaneously by molecular diffusion during rest time or under elevated thermal conditions [9,10,11]. In this respect, the self-healing potential of asphalt can be considered as an eco-sustainable solution strategy to conserve natural resources and relieve environmental pressure. However, in practice, the self-healing capacity of asphalt is weak due to cryogenic conditions and inevitable aging. Therefore, improving the self-healing capacity of asphalt is necessary. In recent years, the rejuvenator encapsulation method, which aims to enhance asphalt healing ability, has drawn researchers’ attention. It can enhance the microcrack repair ability of asphalt concrete and regenerate the aging asphalt binder in place due to the timely replenishment of the healing agent, which represents a hopeful premaintenance approach for asphalt pavements in the future [12,13].

The capsules with different asphalt rejuvenators are the common form of encapsulation to boost the auth-healing capability of asphalt [14,15]. The core-shell microcapsule (μm size) [16,17] and muti-chamber capsule (mm size) [18,19,20] are the primary storage mediums for asphalt rejuvenators. The microcapsules with a rejuvenator showed excellent healing enhancement for asphalt binders in fracture recovery tests [16,21]. However, asphalt concrete with microcapsules cannot effectively enhance the healing ratio of the crack due to limited rejuvenator content and an unsustainable release mode. The muti-chamber calcium alginate capsules showed obvious gradual rejuvenator release potential and provided a sustainable healing capability for asphalt concrete under external cyclic loading [22,23,24]. Hence, incorporating calcium alginate capsules containing a rejuvenator into asphalt concrete will be an ideal treatment strategy for sustainable asphalt pavement.

It is remarkable that the current self-healing experiments on asphalt mixtures mixed with Ca alginate capsules are performed at an idealized temperature condition. The healing capability of asphalt concrete containing capsules depends to a large extent on the capillary flow of the rejuvenator in the microcrack zone, which is closely related to environmental temperature. In order to obtain high healing levels, most of the relevant research tested the healing capacities of the specimens at a moderate test temperature (20 °C) [25,26,27,28,29,30,31,32]. The temperature for capsule activation (healing agent released from Ca alginate capsules via the external compressive loading) was set at 20 °C. Besides, the temperature of healing process was also set at 20 °C. In general, the test temperature does not match the ambient temperature of the actual road. The ambient temperature of asphalt pavement cannot always maintain 20 °C in the actual service environment. The fluctuation of the service temperature has an impact on the healing of asphalt concrete. In cold conditions, the capillary flow speed of the healing agent is slow around the microcrack zone, which decreases the healing rate of asphalt concrete.

Regarding the extent of information available to our knowledge, research concerning the low service temperature (low induction release temperature of the rejuvenator and low healing temperature of the test asphalt mixture specimen) of Calcium alginate capsules embedded in bituminous concrete has yet not been conducted. Even though cracks usually occur in asphalt concrete during a cold period, there are few studies that focus on the crack-healing ratio of bituminous concrete with capsules under low-temperature service conditions. Therefore, this paper focused on the self-healing performance of asphalt concrete with Ca alginate capsules under simulative low-temperature service conditions. Firstly, the Ca alginate capsules were synthesized based on the hole-coagulation bath technique. Secondly, the performance experiments were performed to assess the main properties of the prepared capsules. Thirdly, the healing ratios of the bituminous concrete mixed with capsules under simulative low service conditions were evaluated by the three-point-bending (3PB) testing and cyclic loading–healing testing. Besides, the rejuvenator release ratios of the capsules within the bituminous concrete were determined by Fourier transform infrared spectroscopy (FTIR). Finally, the glass transition temperature and rheological property of the asphalt binder after loadings under different temperatures were explored via a differential scanning calorimeter and a dynamic shear rheometer, respectively.

## 2. Materials and Experimental Methods

### 2.1. Raw Materials

The synthetic ingredients of the Ca alginate capsules include sodium alginate (SA), calcium chloride (CaCl_2_), and Tween-80, which were provided by Shanghai Sinopharm Chemical Reagent Co., Ltd. (Shanghai, China), and the sunflower oil (healing agent) was provided by Arowana Group Co., Ltd. (Shanghai, China). In this study, the density, penetration, and softening point of virgin asphalt (#70) were 1.034 g/cm^3^, 68 (0.1 mm, 25 °C), and 48.4 °C respectively. The density, viscosity, and flash point of the sunflower oil were 0.935 g/cm^−3^, 0.285 pa·s, and 230 °C, respectively. Sunflower oil can supply light component for aged asphalt and help it restore its original property. Besides, sunflower oil has a distinct peak at 1745 cm^−1^**,** while virgin asphalt has no absorption peak between 1700–1750 cm^−1^ [23,31,33]. Therefore, according to FTIR spectra, this characteristic peak can be used to evaluate the healing agent release ratio of the capsules in bituminous concrete after cyclic loading.

### 2.2. Fabrication of Ca alginate Capsules

**Figure 1:**
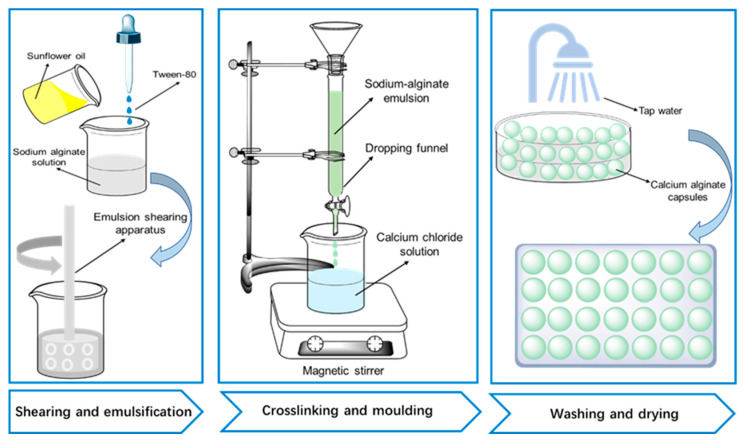
Preparation procedure of Calcium alginate capsules.

The Ca alginate capsules were synthesized at room temperature, and the specific process is shown in Figure 1. The process is divided into four steps: (1) add SA powder to room temperature water and stir for 5 min to form a 2.25 wt% homogeneous SA solution. (2) Add Tween-80 and rejuvenator to the SA solution and shear at 5000 r/min for 15 min to obtain a homogeneous sodium alginate-oil (SA-O) emulsion. The fixed water–oil ratio and surfactant dosage were 1:10 and 5% (oil mass), respectively. (3) Pour the finished emulsion into a custom funnel and instill it into a CaCl_2_ solution (3.0 wt%); this is then kept for 12 h to ensure the complete reaction of the alginate chain with Ca^2+^. (4) Remove the wet capsule, rinse it with water, and leave it in a tray at a moderate temperature for 48 h to obtain a dry capsule. 

### 2.3. Characterization of Ca Alginate Capsules

The characterization tests were performed on the prepared capsules to determine their basic properties. (1) The microstructure inside the capsules was obtained by scanning electron microscopy (Zeiss, Gemini 300, Jena, Germany). The sample preparation process was as follows: (a) the upper and lower parts of the capsules were cut with a blade to obtain a thin section with a thickness of 1 mm; (b) the oil adhered to the surface of the thin section was removed by oil-absorbing paper; (c) the capsule slices were dispersed on conductive carbon adhesive tapes; (d) the sample was sprayed with gold for 30 s. (2) The compression strength of the Ca alginate capsules was determined by the uniaxial compression test (electric compression test machine, ZQ-990, Wuhan, China). Given that the temperature has impaction on the mechanical strength [27,34,35], hence three types of capsules were kept at −15 °C (4 h), 25 °C (4 h) and 160 °C (2 h), respectively, before the test. The capsules were compressively loaded at loading rate with 0.5 mm/min until the occurrence of yield point. In this work, the yield strength was selected as the mechanical strength of capsules. (3) A simultaneous thermal analyzer (STA449F3) with a heating rate of 10 °C/min was used to determine the thermal sensitivity of the capsules. The whole test was conducted under nitrogen atmosphere protection with a heating range of 40−1000 °C. The relative revertant content of capsules was calculated through the remained masses of the blank capsule and rejuvenator.

### 2.4. Preparation of Asphalt Concrete Mixed with Ca alginate Capsules

**Figure 2:**
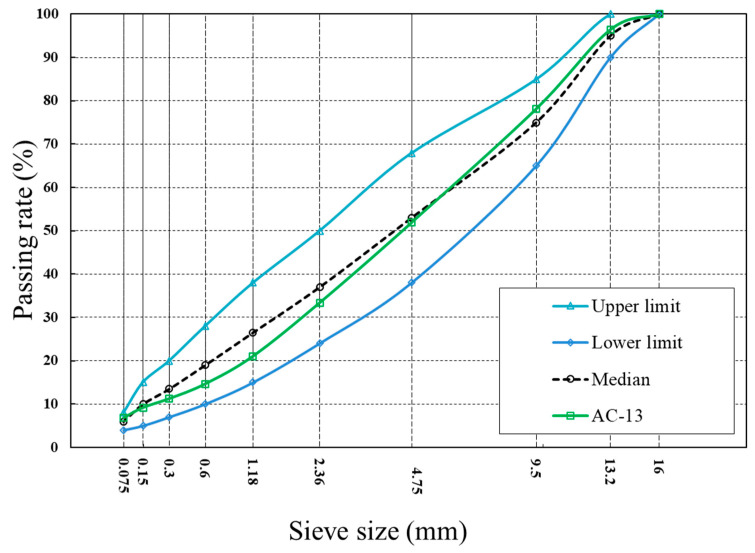
Gradation curve of the asphalt mixture. Reprinted with permission from Ref [36]. 2021, Elsevier.

A dense asphalt mixture [24,36] was selected in this work, and the relevant gradation (AC-13) is presented in Figure 2. The Ca alginate capsules were placed into the bituminous concrete at the end of the mixing procedure, and the weight of the capsules was 0.5% over the total mass of the mixtures. After the mixing procedure, standard rutting noncapsule and capsule asphalt concrete plates were prepared via rutting-slab-forming equipment. Finally, asphalt mix beams (98 mm × 45 mm × 50 mm) were cut from the bituminous concrete slabs, and a notch (10 mm × 4 mm) was cut in the middle zone.

### 2.5. Healing Evaluation of Bituminous Concrete with Ca Alginate Capsules

**Figure 3:**
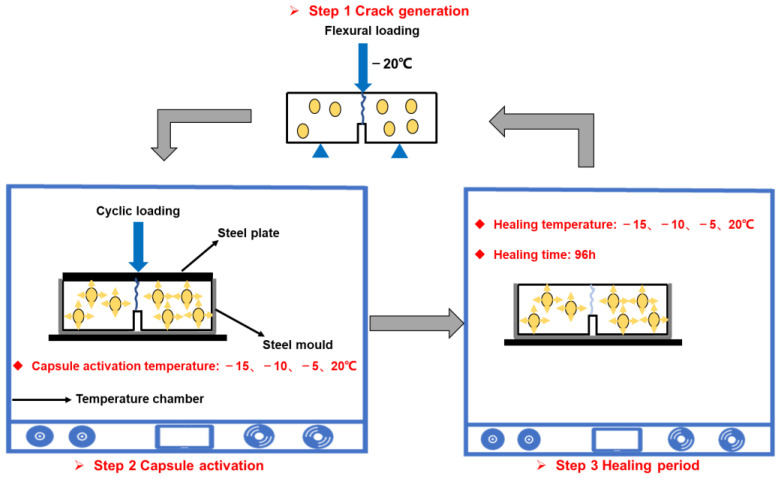
The fracture-healing-refracture test procedure for a bituminous concrete beam.

As illustrated in Figure 3, the fracture-cyclic loading-healing-refracture test was divided into four steps: (1) to obtain the initial bending strength, 3PB tests were performed sequentially on the asphalt mixture beams with and without capsules. The trial temperature was set as −20 °C, and the load conduction rate was set as 0.5 mm/min. (2) The cracked beam was placed into a steel mold, and the ballast pressure (0.7 MPa) was uniformly dispersed by placing steel plates on the beam. The loading cycles were set to 0, 16,000, 32,000, 48,000, and 64,000 to simulate actual vehicle tire loading on the capsules within an asphalt road for a period of 1 year, 2 years, and 4 years, respectively [37]. The test temperatures were set to −15, −10, −5, and 20 °C, respectively. The low-temperature service conditions (−15 °C, −10 °C, and −5 °C) were selected by a combination of average temperature data from the cold regions of China and the temperature limit range of the laboratory test apparatus. A service temperature of 20 °C was chosen to maintain consistency with the healing temperatures mentioned in the existing literature and to serve as an ambient temperature reference group for comparative studies of the healing of asphalt concrete under low-temperature service conditions. External loading was used to cause the capsule to release the encapsulated rejuvenator. (3) After the completion of cyclic loading, the beams in the steel molds were put in thermostats at −15, −10, −5, and 20 °C, respectively, and left to rest for 96 h to recover their strength. (4) At the end of the healing period, the 3PB test (as described in step 1) was conducted on the healed beams again.

The healing rate of above tested bituminous concrete was determined by the strength restoration rate (HI_S_). As shown in Equation (1), the HI_S_ was defined as the specific value between the beam’s flexural strength after the rest period (F_2_) and the initial flexural strength (F_1_). In this trial, three specimens were tested in each group of tests to obtain the average healing rate.

(1) $${\mathrm{HI}}_{S}=\frac{F_{2}}{F_{1}}$$

### 2.6. Quantification of the Healing Agent Released from the Capsules at Different Service Temperatures

**Figure 4:**
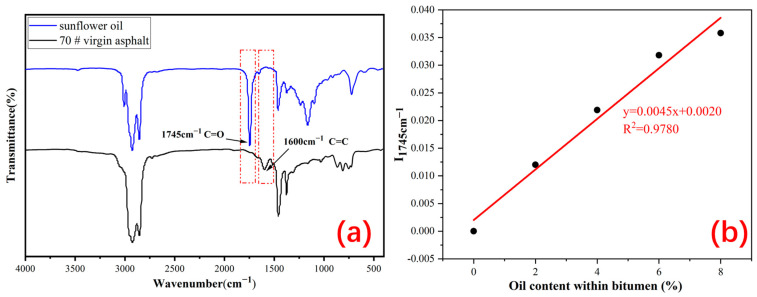
(**a**) Infrared spectra of virgin asphalt and sunflower oil and (**b**) the standard curve between I_1745cm^−1^_ and the rejuvenator content within the virgin asphalt.

In this work, the chemical evaluation of the healing agents released by the capsules in the asphalt mixtures was performed by FTIR spectroscopy (Thermo Fisher Scientific, Nicolet 6700, Waltham, CT, USA). Sunflower oil has a peak at 1745 cm^−1^ with a peak area lined with the oil content in asphalt, while asphalt has no peak in this characteristic range [23,33]. Figure 4a shows the infrared spectra of the oil and asphalt used in this study. Thus, an area of 1745 cm^−1^ can be used to determine the release ratio of the healing agent inside the capsule after compression loading.

The samples of asphalt containing rejuvenator (0, 2, 4, 6, and 8% of asphalt mass) were prepared by mixing oil with asphalt and stirring at 120 °C for 40 min. The prepared asphalt samples were subjected to FTIR tests to obtain the relationship between the 1745 cm^−1^ area and the sunflower oil content in the asphalt binder. Rao et al. found that the peak index in equation (2) could be used to determine the rejuvenator content in the asphalt binder [38].

Figure 4b presents the correlation curve between I_1745cm_^−1^ and the oil content of asphalt. In this work, Origin software was used to find the relationship between the peak area index and the healing agent content of the asphalt, and the linear fitting equation of the peak area index and oil content was derived.

(2) $$I_{1745cm^{-1}}=\frac{The\;peak\;area\;of\;1745cm^{-1}\;}{\Sigma Area\;of\;spectral\;bands\;between\;2000\;and\;600\;cm^{-1}\;\;}$$

After the cyclic loading-healing procedure, the trial beams were put in an oven and heated at 70 °C for 30 min, and the capsules inside the asphalt mix were removed by hand. The loose asphalt mixtures were dissolved in trichloroethylene for 48 h, and then the extracted supernatant was put in a fuming cabinet for 24 h to allow the solvent to evaporate. We added 0.1 g of asphalt into a centrifuge tube and dissolved the asphalt with 2 mL CS_2_. The prepared asphalt oil was poured onto KBr wafers and dried to form a layer of asphalt. The FTIR experiments were conducted within a mid-infrared wave number (400~4000 cm^−1^). The resolution and total scan period were set as 4 cm^−1^ and 64, respectively.

### 2.7. Differential Scanning Calorimetry (DSC) Test of the Extracted Asphalt Binders

**Figure 5:**
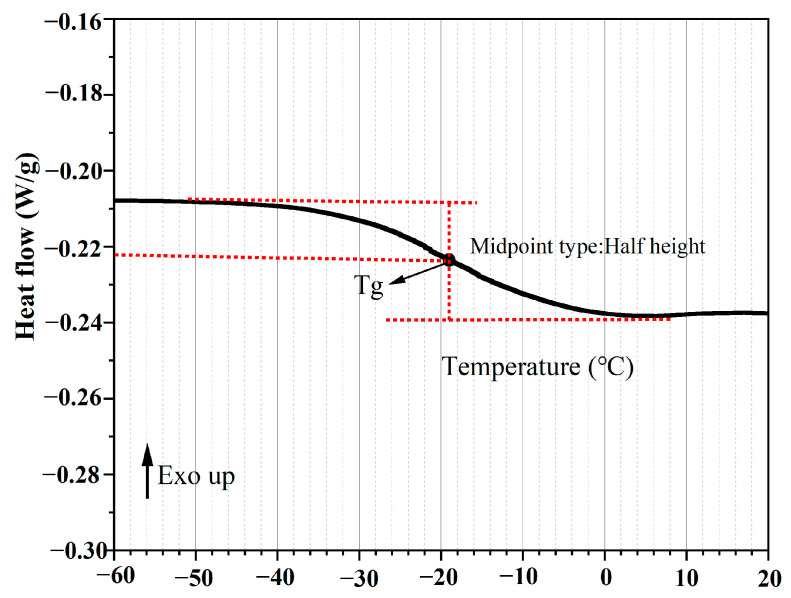
The curve of the glass transition temperature of the asphalt binder.

It has been confirmed that the addition of the rejuvenator into the asphalt binder decreases its glass transition temperature and reduces the modulus of asphalt at a low temperature [39,40,41,42,43]. Hence, the DSC test was performed on different extracted asphalt binders after 64,000 cycles of loading under different test temperatures (−15, −10, −5, and 20 °C) via a TA instrument (TA-DSC2500, New Castle, PA, USA) to determine their glass transition temperatures (Tg). The specific test conditions were as follows: (1) temperature range: −50~160 °C; (2) heating ratio: 10 °C/min; (3) protect atmosphere: N_2_ (flow rate: 50 mL/min). In this work, according to the midpoint method (as presented in Figure 5), the Tg of asphalt binder was obtained.

### 2.8. Rheological Property Testing of the Extracted Asphalt Binders

In order to characterize the rheological performance of the extracted asphalt binders after compressive loading under different temperatures, temperature scan tests were performed by a dynamic shear rheometer (DSR) (Smart Pavement 102) provided by Anton Paar Instruments, Ltd. After 64,000 cycles of compressive loading under different test temperature (−15, −10, −5, and 20 °C), the extracted asphalt binder was selected as test samples. The temperature of the DSR test was from −15 °C to 20 °C. The strain was 0.5%, and the frequency was 10 rad/s. The diameter of the rotor was 8 mm.

## 3. Results and Discussion

### 3.1. Basic Property of the Polymer Capsules

**Figure 6:**
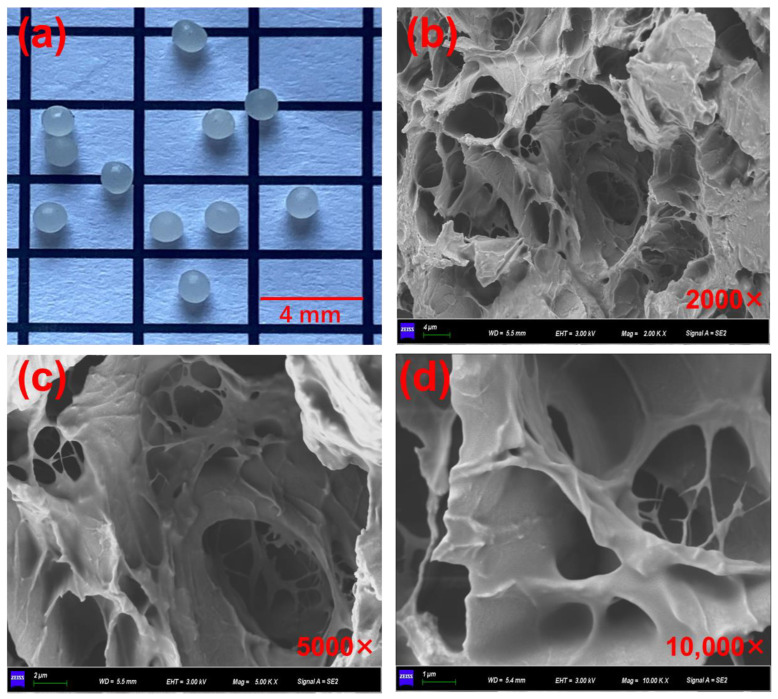
(**a**) Appearance of calcium alginate capsules and (**b**–**d**) the interior structure of the capsules with different magnification times.

Figure 6 presents the morphology and interior structure of the calcium alginate capsules. As can be observed from Figure 6a, the capsules showed near sphere shape, and the average diameter was 1.7 mm. Hence, the capsules can be put into bituminous mixtures as part of the fine aggregates. Besides, as can be observed from Figure 6b–d, the capsule presented a multicavity inter structure. The healing agent droplets were stored in disjunctive cavities with different sizes and shapes. The unique storage mode may make the capsule release its inner healing agent gradually under the action of external loading.

**Figure 7:**
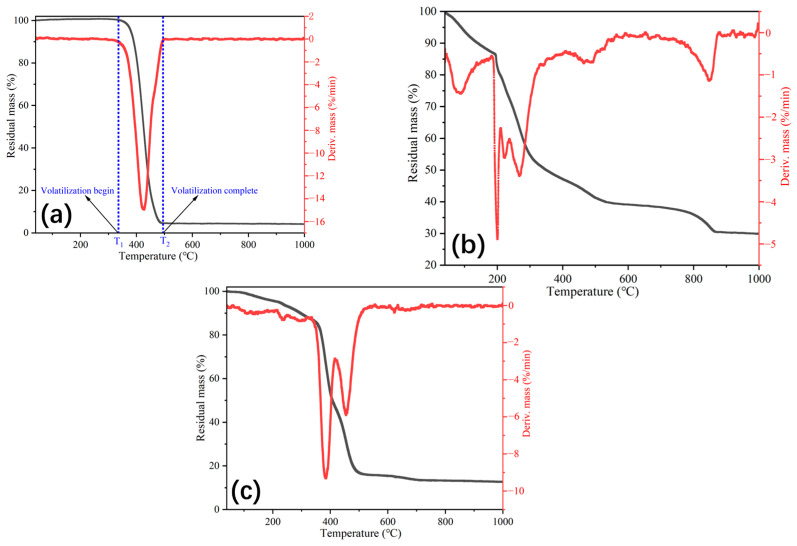
The mass loss of different materials. (**a**) Healing agent, (**b**) blank Ca alginate capsule without the healing agent, and (**c**) Ca alginate capsule containing the healing agent.

The mass losses of the rejuvenator, the blank Ca alginate capsules (without the healing agent) and the Ca alginate capsules containing the healing agent are presented in Figure 7. As shown in Figure 7a, the healing agent (sunflower oil) starts to volatilize at 345 °C and is completely volatilized at 498 °C. Hence, the remaining mass of the oil capsules and blank capsules in this temperature interval (345~498 °C) can be used to obtain the capsules’ actual healing agent content. Figure 7b shows the retained mass of the blank Ca alginate capsules, and their residual mass at 345 °C and 498 °C were 50.1% and 40.9%, respectively. Figure 7c presents the retained mass of the Ca alginate capsules with the healing agent. From 100 °C to 180 °C, the residual mass of the capsule decreased stably due to the vaporization of the moisture and the destruction of the minor glycosidic bonds that exist in the alginate chains. It is noteworthy that the mass loss of the capsules with the rejuvenator was 4.3% at 200 °C (above the fabrication temperature of asphalt mixtures); the capsules have favorable thermal stability at the asphalt mixture production temperature. Besides, the residual masses of the capsules at 345 °C and 498 °C were 86.2% and 17.1%, respectively. Hence, the relative healing agent content of the capsules can be calculated based on formulas (3) and (4). The relative rejuvenator content of the capsules was 58.5%.

(3) $$\varepsilon +\left(\alpha_{1}-\alpha_{2}\right)\left(1-\varepsilon \right)=\beta_{1}-\beta_{2}$$

(4) $$\varepsilon =\frac{\beta_{1}-\beta_{2}+\alpha_{2}-\alpha_{1}}{1+\alpha_{2}-\alpha_{1}}$$

where ε is the relative healing agent content in the capsule (%), $\alpha_{1}$ and $\alpha_{2}$ are the retained mass of the blank capsules without the healing agent at 345 °C and 498 °C, respectively, and $\beta_{1}$ and $\beta_{2}$ are the retained mass of the capsules with the healing agent at 345 °C and 498 °C, respectively.

**Figure 8:**
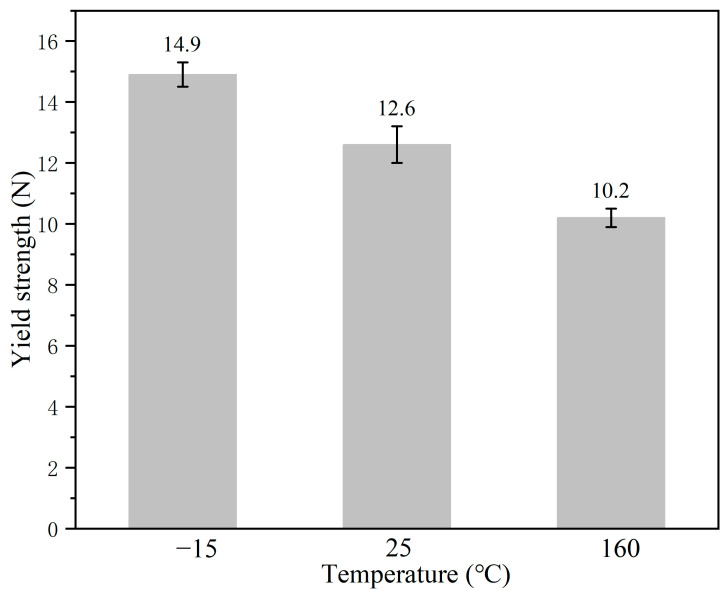
Compressive strength of the Ca alginate capsules.

The capsules (mm size) are designed to be added to bituminous mixtures as part of the fine aggregates. Therefore, the Ca alginate capsules must withstand the mechanical action created in the production period of the asphalt concrete. Practice has shown that the mechanical strength of the capsules used for bituminous mixtures must be greater than 10 N [33,34]. Figure 8 presents the compressive strength of the capsules after specific temperature treatment. The mechanical strength of capsules after different treatments (0, 25, and 160 °C) were 14.9 N, 12.6 N, and 10.2 N, respectively. Lowering the temperature reduces the ability of the calcium alginate molecule chains to move, thus reducing the deformability of the calcium alginate capsules; hence, the capsule has higher strength at lower temperatures. The calcium alginate capsules softened with the increase in temperature, decreasing the compressive strength slightly [27]. The strength of the capsules under 160 °C was still above 10 N, which implied that the capsule could survive the production of asphalt concrete.

### 3.2. Oil Release Assessment of the Capsules in Asphalt Concrete after Cyclic Loading at Different Service Temperatures

**Figure 9:**
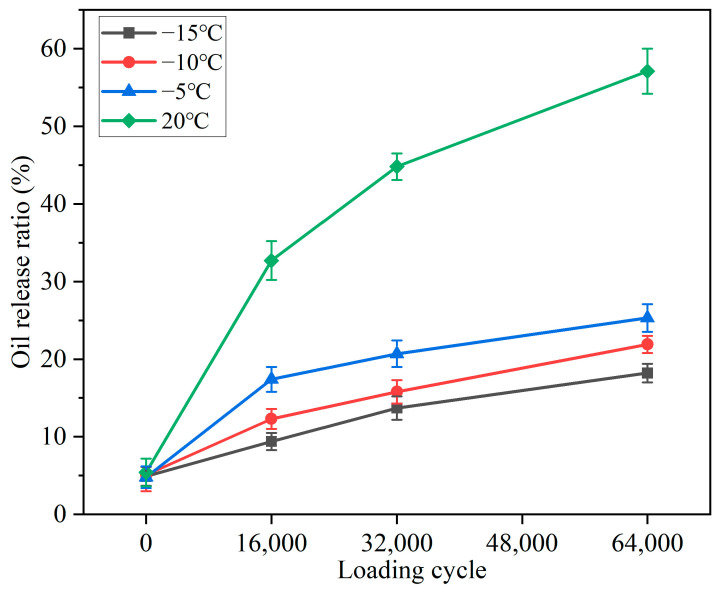
Oil release ratios of Ca alginate capsules within asphalt concrete after loading at different service temperatures.

The oil released by the capsules can soften the asphalt and reduce its viscosity, which enhances the flow ability of asphalt and, thus, improves its crack repair efficiency. Therefore, the healing rate of asphalt concrete is closely dependent on the oil release rate of the capsules presented in the bituminous mixtures. Figure 9 shows the oil release ratio of the capsules in the specimen beams after cyclic loading at different service temperatures. When no external loading was applied on the asphalt concrete beams, the oil release ratios of the capsules after the constant healing period at −15, −10, −5, and 20 °C were 4.9, 5.1, 4.8, and 5.4%, respectively. It could be seen that there were no differences between the oil release ratios, which implied that the service temperature had no influence on the release of the oil in the capsules within the bituminous mixtures without the action of external loading. The premature leakage of oil from the asphalt concrete during the production period was the result of a combination of thermal and mechanical action. Under specific service temperatures, the capsules’ oil release ratio rose with the loading cycles, which implied that the oil release of the capsule needed to be triggered by external loading and that the capsule could gradually let out the encapsulated oil under cyclic loading. The capsule’s own gradual release pattern is based on the unique multi-chamber. The oil was stored in the separated chambers, and the capsule gradually released the healing agent by elastic contraction–expansion under cyclic loading [13,27].

After loading with specific cycles, the oil release ratio of the capsules at 20 °C was obviously higher than that of the capsules at low temperatures ( −15, −10, and −5 °C). The oil release ratio of capsules improved with the increase in service temperature (loading applied temperature and healing temperature). For instance, after compression loading with 64,000 cycles, the capsule oil release ratios at 15, −10, −5, and 20 °C reached 18.2, 21.9, 25.3, and 57.1%, respectively. This interesting phenomenon was consistent with the result reported by Al-Mansoori [44]. The stiffness of the bituminous mixture beams improved with the reduction in test temperature, with the deformation resistance becoming stronger, which made the capsules in the test beams subject to mild compaction. Besides, the strength of the capsules increased with a reduction in temperature, which was proven by the mechanical strength results of the capsules under different temperatures, as shown in Figure 8. Under constant loading cycles, the capsule became stiffer with the reduction in temperature and, thus, more difficult to deform, which made the capsule release less of the rejuvenator.

It was clear that the release of the rejuvenator from the capsules was triggered by the external loading. For pavement areas that cannot be reached by traffic loads, microwave heating can be used to actively induce the release of the healing agent in the capsule at the right time to accelerate the crack healing of asphalt concrete [36].

### 3.3. Selfhealing Performance of Asphalt Concrete Beams under Different Service Temperatures

**Figure 10:**
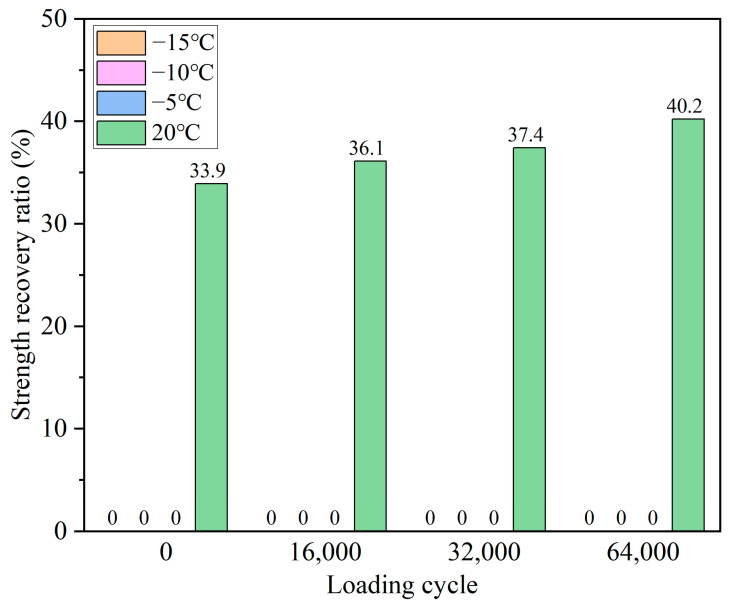
Healing ratio of non-capsule asphalt concrete beams after compression loading at different service temperatures.

**Figure 11:**
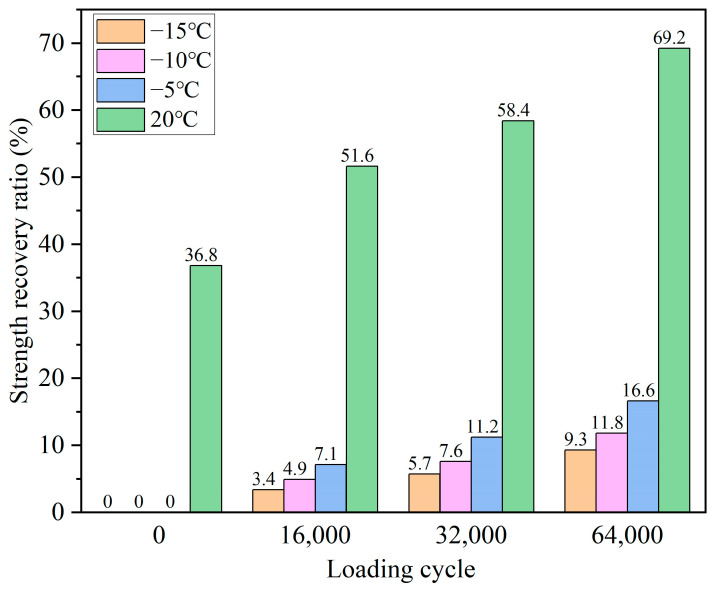
Healing ratio of capsule asphalt concrete beams after compression loading at different service temperatures.

Figure 10 and Figure 11 present the healing ratios of the bituminous mixture beams with and without the capsules after cyclic loading at different service temperatures. As can be seen from Figure 10, the fractured specimen beams without capsules could not obtain any strength recovery after different cycles of loading under −15, −10, and −5 °C, respectively. The reason is that the asphalt was rigid at low temperatures, and the asphalt around the crack zone failed to flow and diffuse; hence, the fractured beams were hard to heal. The weak healing ability of the noncapsule bituminous mixture at a low temperature was also confirmed by the literature [44,45]. When the test temperature was 20 °C, the noncapsule beams had partial strength restoration stemming from the inherent healing property of asphalt at moderate temperatures. Besides, the selfhealing ratios of the noncapsule beams improved slightly with the increase in the loading cycles. The healing ratios of the noncapsule beams were 33.9, 36.1, 37.4, and 40.2%, respectively, after 0, 16,000, 32,000, and 64,000 cycles of loading. The cyclic loading caused the gradual compaction of the aggregate inside the asphalt mixture beam, which caused a slight width reduction between the two surfaces and improved the strength restoration rate of the nonencapsulated beam.

It can be observed from Figure 10 that with zero cyclic loadings applied to the asphalt concrete containing the capsules, the level of healing was 0% at −15, −10, and −5 °C, respectively. Although there was a tiny amount of rejuvenator in asphalt mixture beams, the viscosity of the asphalt was still high (hard to flow). When cyclic loading was performed on the beams with the capsules, they all gained partial strength recovery at low temperatures. The healing ratios all increased with the loading cycles but were still in a low range. When compared to the healing ratios of the non-capsule asphalt beams, it could be inferred that the released healing agent may aid in the recovery of strength at low temperatures after cyclic loading. Due to the limitation of the low temperature, the viscosity reduction effect for the rejuvenator (sunflower oil) was limited. Besides, when the cyclic loading was fixed, the healing ratio of the asphalt concrete beams with capsules all improved with the increase in service temperature. For example, after 64,000 cycles of loading, the healing rates of the asphalt concrete beams with capsules were 9.3, 11.8, and 16.6% at −15, −10, and −5 °C, respectively. When the loading cycles equaled 64000, the oil release rates of the capsules at −15, −10, and −5 °C were 18.2, 21.9, and 25.3%, respectively. The oil released and service temperature jointly affected the healing capability of the asphalt concrete beams with the capsules, and a higher oil release ratio and service temperature gave the beams higher healing ratios.

When the trial temperature was 20 °C, the healing rates of the capsule beams were all significantly higher than those of the beams at low temperatures. The healing ratio of capsule beams improved significantly with the increase in loading cycles. After 0, 16,000, 32,000, and 64,000 cycles of loading, the strength recovery rates of the capsule concrete beams were 36.8, 51.6, 58.4, and 69.2%, respectively, which were higher than those of the noncapsule concrete beams. The improvement in the level of healing was attributed to the collective action of more of the rejuvenator being released and an enhanced healing temperature. In the previous literature [22,23,27,29,33,46,47], when the test temperature was 20 °C, the asphalt concrete with the calcium alginate capsules all obtained higher healing ratios than plain asphalt concrete (without capsules).

### 3.4. Analysis of the Glass Transition Temperature (T_g_) of Asphalt with Oil

According to the mechanical state within various temperature ranges, the asphalt binder (as a temperature-sensitive material) can be classified into three states: the glassy state, the viscoelastic state, and the Newtonian liquid state [48,49]. The transition from the glassy to the viscoelastic state is defined as the glass transition, where the temperature at this point is considered the glass transition temperature (Tg). From the point of view of molecular motion, the glass transition of macromolecules corresponds to the critical states of the onset and freezing of chain segment motions. When the temperature exceeds the T_g_, the molecular chain segments in the asphalt begin to show signs of activity, and the molecular chain conformation changes by rotating within the single bond around the main chain, which results in a rapid increase in deformation and a dramatic decrease in the modulus [39,50].

**Figure 12:**
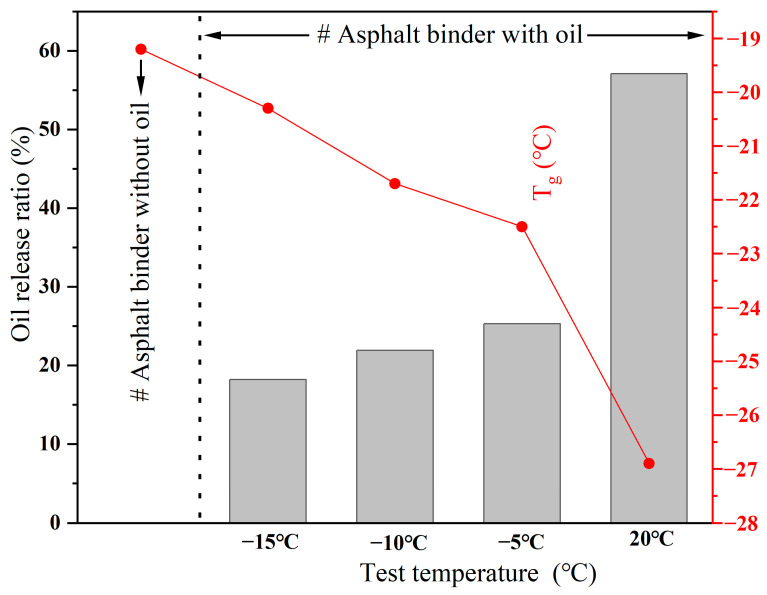
The glass transition temperatures of asphalt after loading with 64,000 cycles under different service temperatures.

The T_g_ of the asphalt binders from the mixture beams after loading with 64,000 cycles at different service conditions are presented in Figure 12. The T_g_ of the asphalt binders with oil was apparently reduced when compared with the asphalt binder without oil. This implied that the release of oil from the capsules could improve the possibility of molecular motions and, thus, decrease the T_g_ of the asphalt. Besides, the healing agent release ratio increased with the rise in the simulated service temperature, and thus, the T_g_ of the asphalt decreased due to the rejuvenation effect of the healing agent, which is consistent with other literature [41,51]. Hence, when compared with a mild temperature (20 °C), the regeneration effect of the healing agent on the asphalt binder under low temperatures (−15, −10, and −5 °C) is less pronounced.

### 3.5. Rheological Property of the Asphalt Binder Containing Oil at Simulated Low-Service Temperatures

**Figure 13:**
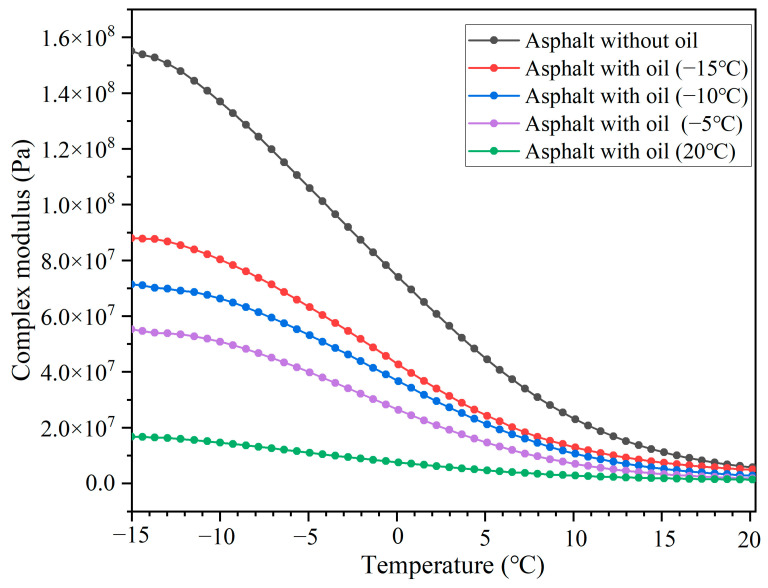
Complex modulus of asphalt binder after loading with 64,000 cycles under different service temperature conditions.

**Figure 14:**
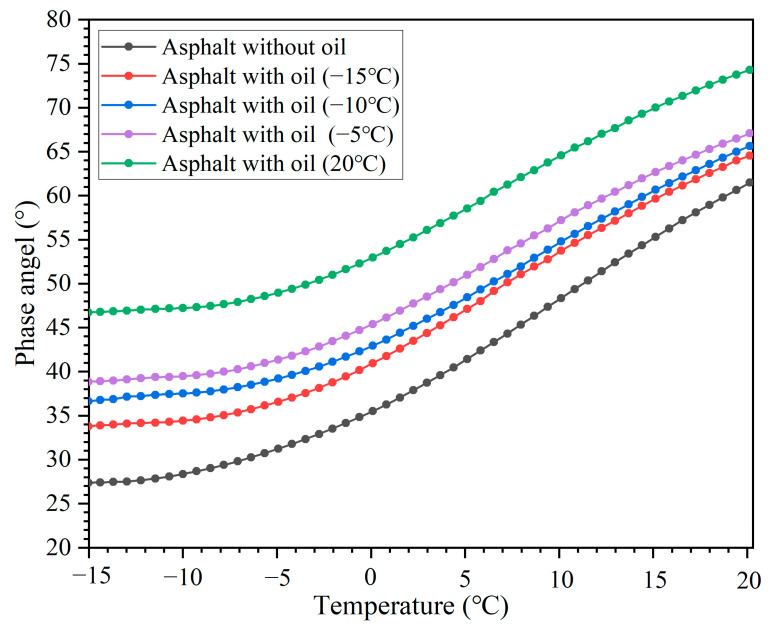
Phase angel of asphalt binder after loading with 64,000 cycles under different service temperature conditions.

Figure 13 and Figure 14 present the complex modulus (G*) and phase angel (δ) of the asphalt binder after 64,000 cycles of compressive loading at different temperatures (capsule activation temperature and healing temperature), respectively. The G* and δ of the asphalt with and without oil all decreased and increased, respectively, with temperature due to the viscoelastic transition of the asphalt binder. When compared to the asphalt without oil, the complex modulus of the asphalt with oil apparently decreased at specific test temperatures. In contrast, the phase angel of the asphalt with oil apparently increased at specific test temperatures. This indicated that the release of oil could change the rheological property of the asphalt binder. When the sunflower oil consists of soft molecules flowing into the asphalt, it could increase the flexible chain ratio of the asphalt and, thus, decrease the complex modulus. Hence, the asphalt with added oil can has better flow ability than the asphalt without oil under the same service temperature, thus having a lower G* and higher δ.

Besides, with a rise in test temperature (from −15 to 20 °C), the G* of the asphalt binder decreased, while the phase angel of the asphalt binder increased. The oil release ratios of the capsules after loading with 64,000 cycles at −15, −10, −5, and 20 °C were 18.2, 21.9, 25.3, and 57.1%, respectively. A higher oil release ratio led to a lower G* and higher δ in the asphalt binder, which indicated that the asphalt binder had better flow ability when servicing at 20 °C than the asphalt binder servicing at low temperatures (−15, −10, and −5 °C). Therefore, when compared with low service temperatures, the asphalt binder within the asphalt concrete containing capsules and serving at a high temperature will have a better softening effect and low-temperature performance due to more oil being released.

## 4. Conclusions

In this work, Ca alginate capsules containing oil were prepared. Evaluation tests were conducted on the capsules to determine their morphological structure, thermal stability, and mechanical resistance. The glass transition temperature and rheological properties of the asphalt binder with and without oil were explored. Besides, the healing release ratio and the level of healing of the capsules in asphalt concrete after loadings under a stimulated low temperature were evaluated. The conclusions are as follows:

(1) The Ca alginate capsules with a mutichamber structure own good thermal stability and compression strength and stratify the production process requirements of asphalt mixtures in a laboratory;
(2) The oil release ratio of the capsules in the asphalt mixture beams increased with the loading cycles. Nevertheless, after loading with specific cycles, the capsules under low-temperature conditions (−15, −10, and −5 °C) had a lower oil release ratio than the capsules at 20 °C;
(3) The release of the oil into the asphalt reduced its complex modulus and increased its phase angle at low service temperatures. Besides, the oil released reduced the glass transition temperature of the asphalt binder and thus enhanced the probability of molecule motion within the asphalt under low temperatures;
(4) The fractured asphalt mixture beams without capsules did not retain any strength recovery at −15, −10, and −5 °C, owing to their reduced inherent healing ability under low temperatures. The incorporation of sunflower oil capsules into the asphalt concrete improved its healing level slightly under low temperatures due to the viscosity reduction effect of the oil. Besides, the healing ratios of the asphalt concrete beams with the capsules under 20 °C were higher than the beam specimens at −15, −10, and −5 °C, respectively.

The asphalt mixture beams with the sunflower oil capsules obtained partial strength recovery (<20%) under a low temperature, and the low healing ability of the asphalt fails to heal the cracks within the asphalt concrete under low-temperature conditions. As asphalt cracking is prone to appear under low temperatures, it is essential to improve the healing ratio of cracked asphalt concrete under low temperatures. Considering the temperature-sensitive properties of asphalt and the viscosity reduction effect of the rejuvenator, the application of the joint action of thermally induced healing and rejuvenator-induced healing may be a promising external healing enhancement strategy for asphalt pavement in cold regions. Microwave absorbing materials can be introduced into asphalt concrete to stimulate heat generation through external microwave heating to promote the flow and diffusion of the rejuvenator, which is expected to solve the problem of the poor healing performance of asphalt concrete under low temperature conditions.

## Data Availability

Data is contained within the article.
